# PRAK Is Required for the Formation of Neutrophil Extracellular Traps

**DOI:** 10.3389/fimmu.2019.01252

**Published:** 2019-06-04

**Authors:** Yan Wang, Yuqing Wang, Jia Wu, Chen Liu, Yu Zhou, Ligu Mi, Yu Zhang, Wei Wang

**Affiliations:** ^1^Department of Immunology, School of Basic Medical Sciences, NHC Key Laboratory of Medical Immunology, Peking University, Beijing, China; ^2^Department of Clinical Laboratory, Peking University People's Hospital, Beijing, China; ^3^Department of Pharmacology, Institute of Materia Medica, Peking Union Medical College, Chinese Academy of Medical Sciences, Beijing, China; ^4^Institute of Biological Sciences, Jinzhou Medical University, Jinzhou, China

**Keywords:** neutrophil extracellular traps (NETs), PRAK, reactive oxygen species (ROS), autophagy, apoptosis

## Abstract

Neutrophil extracellular traps (NETs) are one of the most powerful and specific tools for neutrophils to clean up extracellular microbes, but the mechanisms of NETosis under infection are scarcely studied. In this study, by examining the neutrophils from human peripheral blood and mouse abdomen, we demonstrated that PRAK dysfunction resulted in a significantly reduced NET formation and elevated apoptotic cells. Furthermore, PRAK dysfunction could lead to impaired NET-mediated antibacterial activity and shorten the survival of mice with CLP-induced sepsis. Mechanism studies revealed that attenuated NET formation in PRAK dysfunctional neutrophils correlated with overproduction of reactive oxygen species (ROS), which triggered apoptosis through excessive autophagy. The imbalance of NET formation and apoptosis was further regulated by treatment with lower ROS in hypoxia. Here, we propose a novel candidate, PRAK, which can sense the oxidative stress and regulate the releasing of ROS, may be the master molecular switch to regulate the NETosis-apoptosis axis of neutrophils.

## Introduction

Neutrophils play an important role in innate immunity (1). In the absence of infection, neutrophils have a very short lifespan, and move toward spontaneous apoptosis (2). When pathogens invade, neutrophils perform their function by phagocytosis and degranulation to destroy extracellular pathogens, forming the first line of defense of the immune system (3, 4). A recently established novel antimicrobial function of neutrophils is the formation of neutrophil extracellular traps (NETs) (5). NETs consist of decondensed chromatin associated with granule proteins (5), being a very powerful and specific tool of neutrophils that can trap and kill bacteria (5, 6), fungi (7), parasites (8), etc. However, NETs were suggested as a double-edged sword (9). Excessive NETs are associated with many autoimmune diseases (10), including arthritis (11), systemic lupus (12), anti-neutrophil cytoplasmic antibody (ANCA)-associated vasculitis (AAV) (13), and gout (14). Thus, NET formation, so as called NETosis, needs to be tightly regulated under physiological condition. When NET formation was somehow blocked, apoptosis pathway would be restarted consequently (15). But what triggers NETosis or apoptosis and how to handle the balance between NET formation and apoptosis are still unclear. According to current reports, the factors associated with NET formation include reactive oxygen species (ROS) (16–18), autophagy (15), p38 mitogen activated protein kinase (p38 MAPK) activation (19), histone citrullination (20), etc.

Autophagy has been reported to play a role in regulating the formation of neutrophil extracellular traps. Previous studies have shown that several autophagy inhibitors could hamper Phorbol 12-myristate 13-acetate (PMA)-, lipopolysaccharide (LPS)-induced autophagy-dependent NETs (15, 21, 22). The existence of autophagy inhibitors such as wortmanin would activate caspase, leading neutrophils to apoptosis rather than NETs (15). However, not all results suggested that autophagy accumulation can promote the NETosis. Rapamycin can be used as an autophagy activator, since it can limit the inhibitory effect of mammalian target of rapamycin (mTOR) on autophagy formation through the ULK-Atg13-FIP200 complex (23). It could effectively reduce the LPS-stimulated NET formation, which was not consistent with autophagy inhibitor data (24). However, the role of mTOR in the autophagy pathway was not examined during LPS-stimulated NETs. It is possible that autophagy may have multiple influences on NETs. Apart from its potential direct effect, the indirect effect on NETs by regulating apoptosis and how autophagy controls the balance between NET formation and apoptosis warrant investigation.

Another potential regulator of NETosis is reactive oxygen species (ROS). According to reports, diphenylene iodonium (DPI), an NADPH oxidase inhibitor, could effectively suppress NOX-dependent NETs by inhibiting ROS production (17, 18). But paradoxically, it has also been reported that high concentrations of H_2_O_2_, which provided quite a lot of ROS production, could prevent neutrophils to form NETosis (15). And NOX-independent NET formation is not affected by intracellular ROS generation (25, 26). At current stage, the real influence and tricky mechanism of ROS on NET formation are still unknown.

The third NETosis related factor drawing peoples' attention is p38 MAPK signal. Previous studies have shown p38 MAPK signal could be activated as a downstream of ROS and pretreatment of neutrophils with SB202190 (inhibitor of p38 MAPK) could significantly prevent PMA-stimulated NET formation (19). p38-regulated/activated kinase (PRAK) is the p38 downstream kinase that is likely to be activated by p38 during NET formation(27). Our previous studies have suggested that PRAK could regulate the production of ROS in T cells (data not published) under oxidative stress, protecting cells from apoptosis. Since it can powerfully manipulate the production of ROS, PRAK become a good breakthrough point to explore the role in NOX-dependent NET formation.

To address these questions, the role of PRAK in NET formation and its function of bacterial killing were studied *in vitro* and in cecal ligation and puncture (CLP) model of sepsis. The mechanisms and roles of ROS and autophagy in shaping the biological behaviors of neutrophils were also investigated. The studies demonstrated that the inhibition of PRAK in neutrophils led to decreased NET-mediated extracellular microbial killing and hindered the bacterial removal. Furthermore, our observation indicated that the modulation on PRAK and its ROS releasing may hurt translocation of NE and the accumulation of autophagy by inhibiting the mTOR activation, and finally forced neutrophils to choose apoptosis rather than NETosis.

## Materials and Methods

### Mice

*Prak*^flox/flox^ mice were generated by Shanghai Model Organisms Center (China). *Lyz2*-Cre mice were a gift from Professor Xiaoyu Hu (Tsinghua University). To generate mice with selective *Prak* deficiency in myeloid cells, *Prak*^flox/flox^ mice were crossed with *Lyz2*-Cre mice, both on C57BL/6 background. Ten to twelve-weeks-old *Prak*^flox/flox^ (called “wild-type” here) and *Prak*^flox/flox^ *Lyz2*-Cre (called “knockout” here) mice were littermates and kept in the specific-pathogen-free conditions animal facility of the Peking University Health Science Center. The experimental procedures on use and care of animals were approved by the Ethics Committee of Peking University Health Science Center.

### Isolation and Induction of Human and Mice Neutrophils

Neutrophils were isolated as described previously (28). Briefly, blood samples were collected from healthy volunteers. Neutrophils were isolated from heparinized venous blood by density gradient centrifugation on PolymorphPrep according to the manufacturer's instructions. The purity of the neutrophils was above 95%. Then neutrophils were washed with PBS and suspended in RPMI 1640 supplemented with 4% heat-inactivated fetal bovine serum (FBS). Neutrophils were pretreated with or without PRAK inhibitor (5 μM) for 1 h, and then stimulated with PMA (100 nM) for 4 h at 37°C and 5% CO_2_. The research was approved by the Ethics Committee of Peking University People's Hospital with an approval number of 2016PHB076-01, and performed in accordance with the ethical standards of Declaration of Helsinki.

Briefly, *Prak*^flox/flox^ and *Prak*^flox/flox^ *Lyz2*-Cre mice were injected with sterile 4% thioglycollate in the peritoneal cavity (29). After 8–12 h, neutrophils were enriched (75–80% pure as assessed by flow cytometry using Ly6G and CD11b antibodies). Cells from peritoneal lavage fluid were plated and incubated at 37°C and 5% CO_2_ for 20 min in RPMI 1640 with 0.5% FBS. The cells were washed once with PBS to remove non-adherent cells. And after more enrichment, the neutrophil purity was consistently >90%. Then, isolated neutrophils were stimulated with PMA (100 nM) 12 h.

The percentage of NET formation was manually quantitated by dividing the number of NET-forming neutrophils (expanded nuclei and releasing DNA fibers) by the total number of cells in 5–8 random microscopic fields and multiplying the values by 100 (30).

### Reagents and Antibodies

Phorbol-myristate-acetate (PMA), lipopolysaccharide (LPS), ionomycin and anti-LC3 antibody were purchased from Sigma-Aldrich. Sytox Green Nucleic Acid Stain, and Dihydroethidium (DHE) were from Life Technologies. The anti-p62 antibody, anti- cleave-caspase3 antibody, phospho- and total mTOR, 70 S6 kinase, 4EBP1, and ERK antibodies were all from Cell Signaling Technology. Horseradish peroxidase-conjugated antibody to rabbit IgG or to mouse IgG was from biodragon-immunotech. The anti-neutrophil elastase antibody and anti-myeloperoxidase antibody were purchased from Abcam. Alexa Fluor 488 goat anti rabbit IgG or mouse IgG and Hoechst 33342 were purchased from Zhongshan Golden Bridge Biotechnology. zVAD-fmk was purchased from Selleck Chemicals. The PRAK inhibitor was synthesized by Wuxi Pharma (China).

### Immunofluorescence Microscopy

NETs were analyzed by immunofluorescence microscopy as previously described (31). Neutrophils were placed on glass coverslips in 24-well-plates in presence or absence of PRAK inhibitor, and stimulated with PMA for 4 h and at 37°C and 5% CO_2_. Subsequently, cells were fixed with 4% paraformaldehyde for 2 h and permeabilized with 0.1% Triton X-100 for 5 min at RT. The fixed cells were blocked with blocking buffer (5 mg/ml BSA in PBS) at 37°C for 1 h. Then cells were stained with the primary antibody such as anti-NE, anti-MPO, or anti-LC3 overnight at 4°C. After three washes with PBS, the cells were incubated for another 1 h with secondary goat anti-rabbit IgG or mouse IgG antibody conjugated with Alexa Fluor 488. The DNA was stained with Hoechst 33342 at RT for 5 min. Specimens were captured with confocal microscope.

### Live Cell Imaging

Neutrophils were placed in a glass-bottomed culture dish for live cell observation and incubated with or without PRAK inhibitor for 1 h in the presence of Hoechst 33342 and Sytox green. Then, the cells were stimulated with PMA at 37°C and 5% CO_2_. The fluorescent signals were detected and imaged with a Leica SP5 workstation. Cell morphology was observed using differential interference contrast (DIC). The cells were monitored by autofocus images, and multiple image stacks were captured every 1 min (15, 31).

### Bacterial Killing Assay

The *E.coli* strain BL21 was grown to log phase in Luria Bertani medium at 37°C. Neutrophils were pretreated with or without PRAK inhibitor for 1 h and further stimulated with PMA for 3 h at 37°C and 5% CO_2_ to induce formation of NETs. To block bacterial phagocytosis, neutrophils were incubated with phagocytosis inhibitor Cytochalasin D before infection of bacteria. To inhibit NET-mediated bacterial killing, neutrophils were incubated with DNase prior to the addition of bacteria. Then cells were incubated with bacteria for 1 h. Bacterial cultures were grown on Luria Bertani agar plates. After a 24-h bacterial culture, bacterial counts were determined (5, 18).

### Quantification of ROS Generation

Intracellular ROS was determined using a fluorescent probe, Dihydroethidium (DHE) assay. Neutrophils were pretreated with or without PRAK inhibitor for 1 h and further stimulated with PMA for 30 min. Then, neutrophils were incubated with DHE for 20 min and washed with PBS, followed by measurement of DHE fluorescence with flow cytometry.

### Western Blot Analysis

Western blot analysis was performed on cells stimulated with PMA for 30 min with or without PRAK inhibitor pretreatment. Neutrophils were lysed in cell lysis buffer containing 150 mM NaCl, 50 mM Tris-HCl (pH7.5), 1 mM EDTA, 1% Na-deoxycholale, 1% Triton-100, 0.1% SDS and 10 mM “cocktail” of protease inhibitors. Protein was separated on sodium dodecyl sulfate-polyacrylamide gel electrophoresis (SDS-PAGE) gel and transferred onto a polyvinylidene difluoride (PVDF) membrane. Subsequently, the (PVDF) membranes were probed by incubation with the appropriate primary antibodies at a dilution of 1:500 (or as stated otherwise below) overnight at 4°C, followed by incubation with secondary antibody horseradish peroxidase (HRP)-conjugated antibody to rabbit IgG or to mouse IgG (diluted 1:5,000) for 1 h at room temperature. The protein bands were visualized with a SuperSignal WestPico Kit according to the manufacturer's instructions (Thermo Fisher).

### Cecal Ligation and Puncture (CLP) Sepsis Animal Model

Sepsis was induced by cecal ligation and puncture (CLP) as described previously (32). Briefly, the mice were anesthetized with pelltobarbitalum natricum and shaved an incision in the abdomen. The cecum was exposed and ligated distal to the cecum. The cecum was punctured with an 18-g needle, and a small amount of cecum content was extruded. Then the cecum was replaced into the abdominal cavity, and the incision was closed.

### Cytokine Measurement

The concentrations of mouse IL-6, and TNF-α in the peritoneal lavage fluid after CLP 6 h were measured by ELISA kits (BioLegend) according to the manufacturer's instructions.

### Statistical Analysis

Statistical analyses were performed using SPSS 17.0 and GraphPad Prism 7. Data are presented as the mean ± standard deviation (SD). Two-tailed Student's *t*-test or paired *t*-test were used for single comparison, and two-way ANOVA was used for multiple comparisons. The survival rates of CLP model were analyzed with Mantel-Cox log-rank test. In all tests, *P* < 0.05 were considered statistically significant.

## Results

### PRAK Regulates NET Formation in Neutrophils

**Figure 1:**
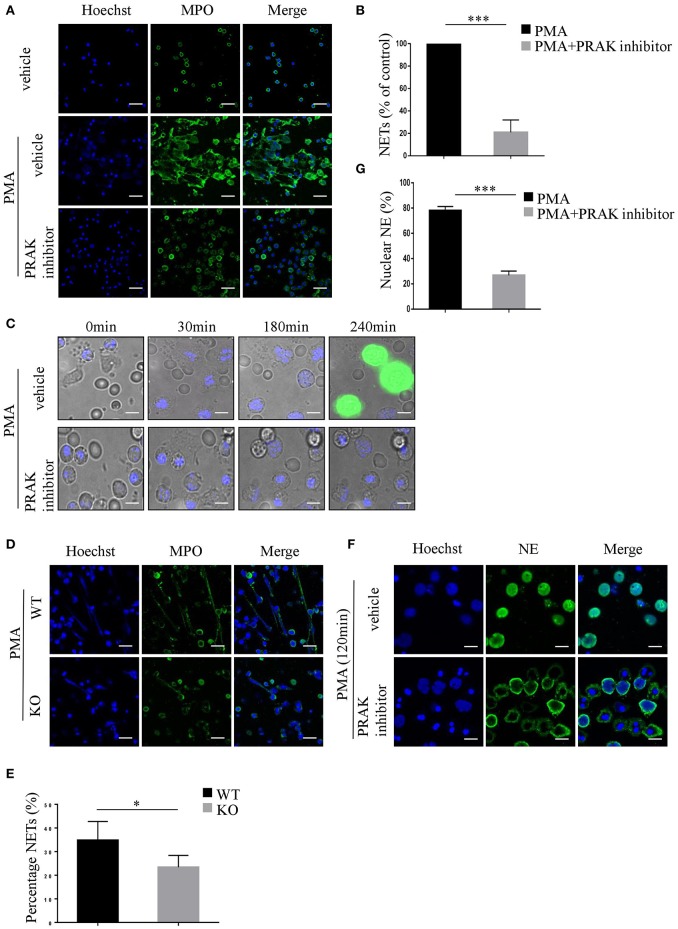
PRAK is required for NET formation. **(A–C,F,G)** Neutrophils were isolated from the peripheral blood of healthy volunteers. **(A)** Neutrophils pretreated with or without PRAK inhibitor (5 μM) were incubated with PMA (100 nM) for 4 h, fixed, and stained with anti-MPO (MPO; green) and DNA (Hoechst 33342; blue). Scale bar = 25 μm. **(B)** NET formation was quantified with fluorescence microscopic analysis. Results are presented as the percentage of control (PMA-stimulated neutrophils) from 5 independent experiments and analyzed by paired *t*-test (^***^P < 0.001). **(C)** PMA-stimulated neutrophils pretreated with or without PRAK inhibitor were monitored by live cell imaging for three parameters: morphology using differential interface contrast (DIC), chromatin using cell-impermeable DNA dye Sytox Green (green) and cell-permeable DNA marker Hoechst 33342 (blue). Important time points are shown in min. **(D,E)** Neutrophils were isolated from the peritoneal cavity of *Prak*^flox/flox^ (WT) and *Prak*^flox/flox^ *Lyz2*-Cre (KO) mice, and NET formation was induced with 100 nM PMA for 12 h. **(D)** NETs were stained with anti-MPO (MPO; green) and DNA (Hoechst 33342; blue). Scale bar = 25 μm. **(E)** Results are shown as the mean percentage of NET forming neutrophils ± SD (*n* = 5, ^*^P < 0.05, paired *t*-test). **(F)** Localization of NE (green) and Hoechst -stained DNA (blue) in neutrophils after PMA- stimulation for 120 min with or without pretreatment of PRAK inhibitor (5 μm). Scale bar = 10 μm. **(G)** Quantitation of the percentage of NE that colocalized with nucleus. The experiments were repeated 5 times and at least 50 cells per group were quantified each time (^***^P < 0.001, paired *t*-test).

As we know, ROS is the main production of NADPH oxidase, which is fundamental for NET formation (17, 18), hence there is a certain possibility that by manipulating ROS production, PRAK may be involved in NETosis. To verify this hypothesis, fresh isolated neutrophils from healthy human blood were treated with or without PRAK inhibitor for 1 h, and then stimulated with PMA for 4 h at 37°C to induce NET formation. Compared to neutrophils stimulated with PMA alone, neutrophils pretreated with PRAK inhibitor showed attenuated levels of NET formation with far less typical DNA filaments and less diffused nuclei (Figure 1A). Based on the morphology (Figures 1A,B) and cell-free DNA (cf-DNA) concentration (Figure S1A), pharmacologic inhibition of PRAK statistically decreased NET formation by about 70% *in vitro* induction. And PRAK inhibitor could exert this obstructive effect on NET formation with a dose-dependent manner (Figures S1B,C) but was not due to its toxicity (Figure S1D). To further examine PRAK's effect on the dynamic process of NET formation, PMA-stimulated neutrophils pretreated with or without PRAK inhibitor were monitored via live cell imaging. By using the cell-impermeable DNA dye Sytox green and the cell-permeable DNA dye Hoechst 33342, we could clearly estimate the status of nucleus transition and the membrane integrity during NETosis (15, 31). In the presence of PRAK inhibitor, 240 min after PMA stimulation, which was the end point of our observation, rarely neutrophils showed Sytox positive but contained pyknotic nuclei, demonstrating the membranes of these cells were still integral and probably they were not in the process of NETosis (Figure 1C). In parallel, majority of neutrophils stimulated with PMA alone concurrently were stained with Sytox green and showed intracellular chromatin decondensation, indicating disintegration of cell membrane and formation of NETs (Figure 1C). Later, we got the similar results from *Prak* knockout mice. Neutrophils from WT mice peritoneal cavity fluid can form clearly web-like structure and elevate MPO expression, while neutrophils from *Prak* knockout mice can hardly form NETosis with the stimulation of PMA (Figures 1D,E). To make a solid judgement, we further examined the translocation of neutrophil elastase (NE) to the nucleus during NETosis, which was the critical step in NET formation (33, 34). We chose to monitor the localization of NE after PMA-stimulation for 2 h with or without PRAK inhibitor pretreatment. In PMA alone group, NE was predominantly found in the nucleus when neutrophils underwent NETosis as reported (Figure 1F; Figure S1E). In contrast, in PRAK inhibitor pretreatment group, NE was excluded from the nucleus (Figure 1F), with < 30% of the total NE in the nucleus 2 h after stimulation (Figure 1G). Taken together, these results suggested that pharmacologic inhibition of PRAK hurt the emigration of the critical enzyme, membrane split and chromatin decondensation and finally hampered NET formation.

### Inhibition of PRAK Decreases NET-Mediated Extracellular Bacterial Killing

**Figure 2:**
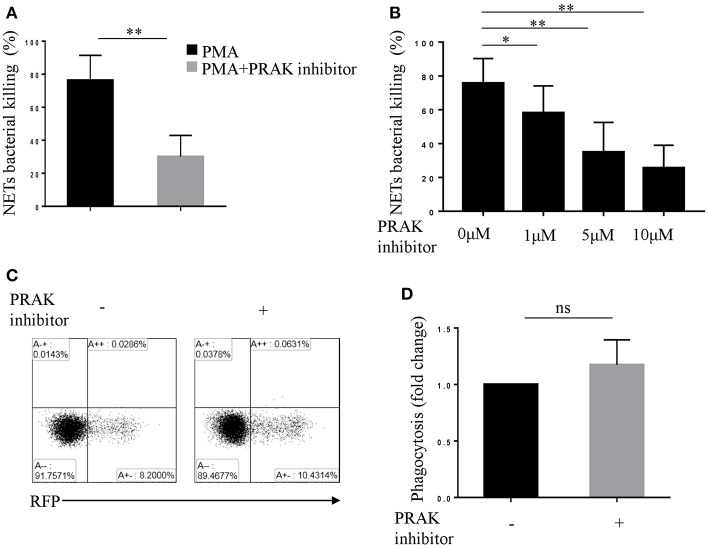
PRAK inhibitor suppresses NET-mediated extracellular bacterial killing. Extracellular NET-mediated bacterial killing by PMA-stimulated neutrophils for 3 h was determined with or without PRAK inhibitor **(A)** 5 μM or **(B)** 0–10 μM pretreatment. Results are shown as the mean percentage of bacterial killing ± SD (*n* = 5, ^*^P < 0.05, ^**^P < 0.01). Paired *t*-test was used for single comparison, and two-way ANOVA was used for multiple comparisons. **(C)** Neutrophils were incubated with *E.coli*-RFP (expression of RFP protein), and the phagocytosis was monitored by flow cytometry according to RFP expression. **(D)** Results are presented as fold change of control (no inhibitor added, *n* = 5, ns, no significant, paired *t*-test).

Neutrophils take the advantage of the sticky web-like characteristic of NETs to disarm and kill a variety of microbes in extracellular (5, 35), therefore NETs are featured as another killing tool of neutrophils, apart from phagocytosis (18, 36). To verify whether PRAK regulation of NETosis did influence NET-mediated extracellular bacterial killing, we performed bacterial killing assays as previously described (5, 18). PMA-stimulated neutrophils were incubated with Cytochalasin D, which blocked phagocytosis without affecting NETs and then infected with *E.coli*. As shown in Figure 2A; Figure S2A, there were large quantities of bacteria left in PRAK inhibitor pretreatment group and hence the percentage of bacterial killing was significantly decreased compared to untreated neutrophils. Moreover, the treatment with various concentrations of PRAK inhibitor resulted in a reduction of NET-mediated extracellular bacterial killing in a dose-dependent manner (Figure 2B; Figure S2B). Since our hypothesis suggested that PRAK dysfunction would specially manipulate NET formation and the other functions of neutrophils such as phagocytosis were supposed not to be affected. To verify it, the effect of PRAK inhibitor on the phagocytic function of neutrophils was then examined. The neutrophils were treated with DNase before infection with *E.coli*, which dismantled NETosis but did not affect phagocytosis. As expected, there was no significant difference in phagocytosis between the PRAK inhibitor pretreatment or not pretreatment groups of cells (Figures 2C,D). This result suggested that PRAK regulated NET-mediated extracellular microbial killing, but had hardly any effect on phagocytic function of neutrophils.

### Impaired NET Formation in PRAK Dysfunctional Neutrophils Results in Enhanced Caspase3 and Autophagy Activation

**Figure 3:**
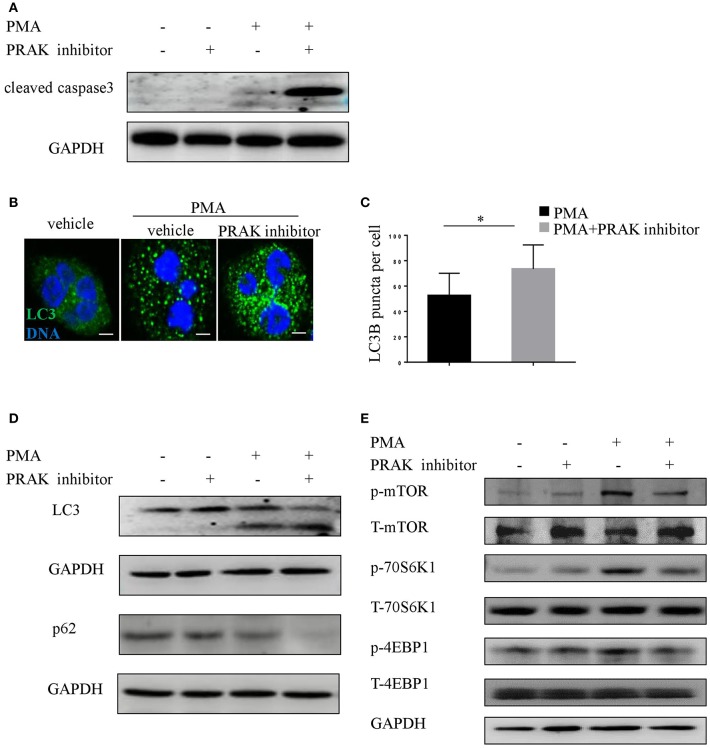
Impaired NET formation in PRAK dysfunctional neutrophils results in enhanced caspase3 and autophagy activation. **(A)** Western blot analysis of cleaved caspase3 in neutrophils stimulated with PMA (100 nM) for 8 h with or without PRAK inhibitor (5 μM) pretreatment. **(B–E)** Neutrophils were pretreated with or without PRAK inhibitor (5 μM) for 1 h, followed by stimulation with PMA (100 nM) for 30 min. **(B)** Cells were stained for DNA (Hoechst 33342; blue) and LC3B (LC3B; green). Scale bar = 5 μm. **(C)** LC3B-positive puncta per cell were quantified using ImageJ. Results are shown as the mean LC3B puncta count ± SD (*n* = 5, ^*^P < 0.05, paired *t*-test). Immunoblotting analysis of the levels of **(D)** autophagy (LC3 and p62) and **(E)** mTOR pathways (mTOR, 4EBP1, and 70S6K).

Series of studies by other groups have demonstrated that NETosis would be interfered if caspase-dependent apoptosis was overactive, and vice versa (15, 37). Therefore, there was a possibility that the effect of PRAK dysfunction on NET formation was on account of its influence on the extent of apoptosis. In fact, the results on caspase-3 expression in PMA plus PRAK inhibitor-pretreated neutrophils revealed a much higher activation of apoptosis (Figure 3A). As we know, mTOR-autophagy pathway was usually in charge of cell apoptosis, we next sought whether the enhanced apoptosis and reduced NETosis by PRAK inhibitor were caused by mTOR signaling or autophagy accumulation. The data showed that PRAK inhibitor-pretreated neutrophils with PMA exhibited clearly higher production of the LC3 fluorescence with a stronger punctate staining pattern than untreated neutrophils (Figures 3B,C). Later, the autophagy formation in response to PMA stimulation was further confirmed by immunoblotting. Although PRAK inhibitor alone would not induce the expression of LC3-II, it would sharply upregulate LC3-II expression upon PMA stimulation (Figure 3D). Similarly, the p62 protein level, as another autophagy activation indicator, was significantly decreased in PRAK inhibitor-pretreated neutrophils compared to that in untreated neutrophils (Figure 3D). As we know, mTOR signal was a master negative regulator of autophagy formation (23, 38), we next examined to verify the enhanced autophagy activation by PRAK inhibitor was determined by the deactivation mTOR. As Figure 3E shown, treatment of neutrophils with PRAK inhibitor resulted in a marked suppression of the phosphorylation of mTOR induced by PMA stimulation (Figure 3E). Additionally, the phosphorylation of 4EBP1 and 70S6K, which are the direct downstream of mTORC1, were decreased in PRAK inhibitor- pretreated neutrophils (Figure 3E). And this regulation on mTOR signal by PRAK inhibitor was specific, since other signaling such as ERK was not varied (Figure S3A) (39). Briefly, these results illustrated that the attenuation of NET formation of neutrophils by PRAK dysfunction may be associated with elevated caspase-dependent apoptosis, owing to over-activity of autophagy formation caused by the enhanced mTOR repression.

### Reducing Apoptosis Rescues NET Formation in PRAK Dysfunctional Neutrophils

**Figure 4:**
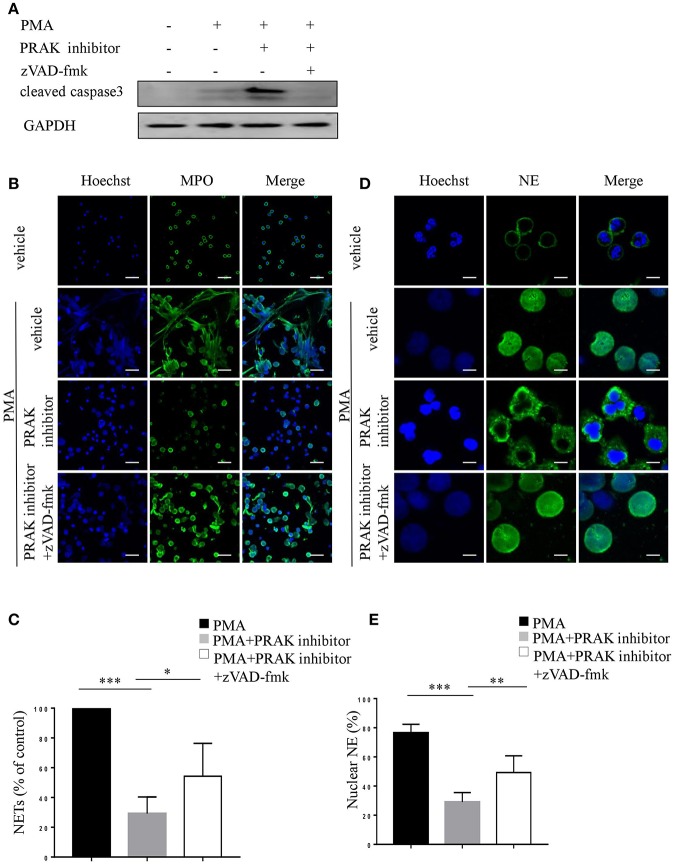
zVAD-fmk rescues NET formation in PRAK dysfunctional neutrophils via reduced apoptosis. Neutrophils were pretreated with or without PRAK inhibitor (5 μM) and further stimulated with PMA (100 nM) in the presence of zVAD-fmk (10 μM) for 8 h **(A)**, 4 h **(B,C)**, or 2 h **(D,E)**. **(A)** Immunoblotting analysis of cleaved caspase3. **(B)** NETs were stained with anti-MPO (MPO; green) and DNA (Hoechst 33342; blue). Scale bar = 25 μm. **(C)** Quantitation of NET formation was via fluorescence microscopic analysis. Results are presented as the percentage of control (*n* = 5, ^*^P < 0.05, ^***^P < 0.001). **(D)** Localization of NE (green) and Hoechst -stained DNA (blue). Scale bar = 7.5 μm **(E)** Quantitation of the percentage of NE that colocalized with nucleus (*n* = 5, ^**^P < 0.01, ^***^P < 0.001). Two-way ANOVA was used for multiple comparisons.

As we shown above, PRAK inhibitor would promote apoptosis and suppress NETosis upon PMA stimulation. To further examine whether the altered NET formation of neutrophils by PRAK inhibitor was actually resulted from its facilitation of apoptosis, we used zVAD-fmk, a pan-caspase inhibitor, to forcedly cut down the apoptosis of neutrophils after PMA stimulation. Indeed, as Figure 4A shown, zVAD-fmk could clearly restrain the activity of caspase 3. Furthermore, the addition of zVAD-fmk could partially rescue the decreased MPO expression and enhance the chromatin decondensation in PRAK inhibitor-pretreated neutrophils during PMA-induced NETosis (Figures 4B,C). In consistent, the percentage of nuclear NE was also increased in the zVAD-fmk-treated group (Figures 4D,E). Hence, these data suggested that the impaired NET formation by PRAK inhibitor could be, at least, partially rescued by reduced apoptosis.

### Rescue of NET Formation in PRAK Dysfunction Neutrophils by Correcting the ROS Production

**Figure 5:**
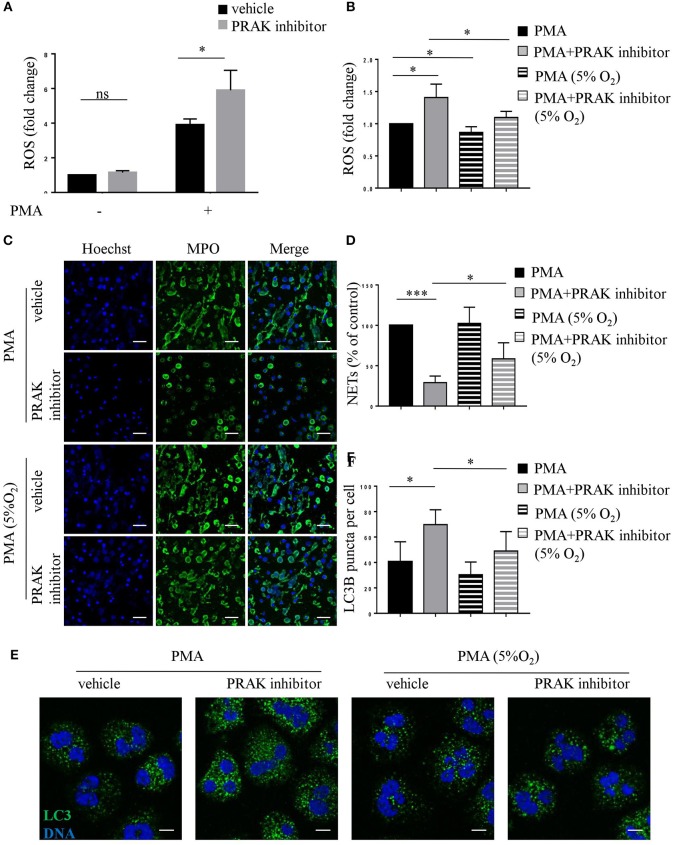
Hypoxic environment (5% O_2_) rescues NET formation in PRAK dysfunctional neutrophils. Neutrophils were stimulated with PMA (100 nM) for 30 min **(A,B,E,F)** or 4 h **(C,D)** after a 1-h pretreatment with or without PRAK (5 μM) inhibitor under normoxia or hypoxia conditions. **(A,B)** Quantification of ROS. Results are presented as fold change of control (*n* = 5, ns, no significant, ^*^P < 0.05). **(C)** NETs were stained with anti-MPO (MPO; green) and DNA (Hoechst 33342; blue). Scale bar = 25 μm. **(D)** Quantitation of NET formation was via fluorescence microscopic analysis. Results are presented as the percentage of control (*n* = 5, ^*^P < 0.05, ^***^P < 0.001). **(E)** Cells were stained for DNA (Hoechst 33342; blue) and LC3B (anti-LC3B antibody; green). Scale bar = 7.5 μm. **(F)** Results are shown as the mean LC3B puncta count ± SD (*n* = 5, ^*^P < 0.05). Paired *t*-test was used for single comparison, and two-way ANOVA was used for multiple comparisons.

Our previous research has shown that PRAK could regulate intracellular ROS generation to reduce oxidative damage (data not published). As shown by flow cytometry analysis, PRAK inhibitor-pretreated neutrophils showed increased production of ROS in response to PMA stimulation compared to the control cells (Figure 5A). Due to the possibility that excessive ROS was the main reason for high caspases expression and apoptosis (40), we tested the apoptosis and NETosis of neutrophils in PRAK inhibitor pretreatment after rectifying their ROS accumulation in a hypoxic environment (5% O_2_). Compared to the neutrophils stimulated with PMA under normoxic PO_2_, neutrophils showed a mild decreased intracellular ROS content in response to PMA stimulation under hypoxic condition but it can surely reduce the elevated ROS caused by PRAK inhibitor (Figure 5B). From our data, hypoxic environment (5% O_2_) could rescue the release of the web-like structures composed of decondensed chromatin and MPO in PRAK inhibitor-pretreated neutrophils during PMA-induced NETosis (Figures 5C,D). Furthermore, as Figures 5E,F shown, reduced ROS production in 5% O_2_ could restrain the over-activity of autophagy in PRAK inhibitor-pretreated neutrophils during PMA stimulation and may partially rescue the apoptosis. Consistently, the attenuation of NET-mediated bacterial killing and NE translocation of neutrophils by PRAK dysfunction could be also rescued by treatment with lower ROS in hypoxia (Figure S4). In addition, PRAK inhibitor could also inhibit LPS-stimulated NOX-dependent NETs (Figures S5A,B). Since ROS was firmly related to NADPH oxidase and theoretically ROS level will not interfere with NOX-independent NETs, to examine whether PRAK specifically affects the formation of NOX-dependent NETs, we also examined the effect of PRAK inhibitor on ionomycin-induced NETs. As expected, the suppressive effect of PRAK inhibitor on NOX-independent NETs was not observed (Figures S5C,D). Briefly, these data above suggested that the rectification of ROS and autophagy may rescue the defective neutrophil NETosis brought by PRAK inhibitor.

### PRAK Deficiency Blocks NET Formation of Neutrophils in a Murine Sepsis Model

**Figure 6:**
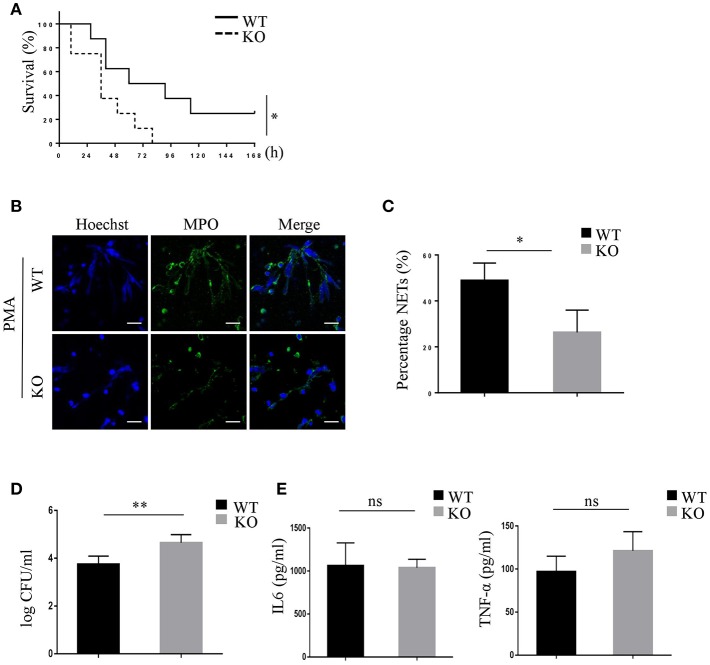
PRAK deficiency shortens survival in mice with CLP-induced sepsis via decreased NET formation. **(A)** The survival rates of *Prak*^flox/flox^ (WT) and *Prak*^flox/flox^ *Lyz2*-Cre (KO) mice after CLP were evaluated over 7 days (*n* = 8, ^*^P < 0.05). **(B)** Neutrophils were isolated from the peritoneal cavity of *Prak*^flox/flox^ (WT) and *Prak*^flox/flox^ *Lyz2*-Cre (KO) mice after 6 h CLP, and NET formation was induced with 100 nM PMA for 12 h. NETs were stained for myeloperoxidase (MPO; green) and DNA (Hoechst 33342; blue). Scale bar = 25 μm. **(C)** Results are shown as the mean percentage of NET forming neutrophils ± SD (*n* = 5, ^*^P < 0.05, paired *t*-test). **(D)** Quantification of bacterial load in peritoneal lavage fluid of mice 6 h after CLP (*n* = 5, ^**^P < 0.01, *t*-test). **(E)** IL-6 and TNFα levels in peritoneal lavage fluid of mice 6 h after CLP measured by ELISA (*n* = 5, ns, no significant, *t*-test).

To investigate whether PRAK would influence the NETosis and killing function of neutrophils during bacterial infection, the mouse CLP-induced sepsis model was used to measure the bacterial cleaning of neutrophils (41). As Figure 6A shown, Mantel-Cox log-rank survival analysis for the WT and *Prak* knockout mice after puncture in the caecum illustrated that the *Prak* knockout mice had a lower overall survival capacity (Figure 6A). *Prak* knockout mice had died very fast and all mice in the KO group died within 4 days, while 25% mice (2 in 8) in the WT group still survived for 7 days (Figure 6A). Moreover, we analyzed NET formation in the neutrophils from septic mice. 6 h after CLP, neutrophils were isolated and purified from the mouse peritoneal lavage fluid and stimulated with PMA for 12 h. Due to the bacterial infection, WT neutrophils were pretty active and produced a huge amount of NETosis upon PMA stimulation with large nuclear areas and plenty of released DNA fibers (Figures 6B,C). In comparison, barely NET formation was observed in neutrophils from *Prak* knockout mice (Figures 6B,C). Likewise, the serum concentrations of cf-DNA (NETs) were higher in WT mice than that in KO mice (Figure S6A). To further assess the effects of NETs capacity on impeding bacterial spreading during sepsis, the bacterial load was examined in the peritoneal cavity. Compared to WT mice, amount of bacterial load was observed in the peritoneal cavity of knockout mice and could not be cleaned up by these *Prak* knockout neutrophils (Figure 6D). Notably, PRAK deficiency specifically harmed the NETosis in neutrophils but did not affect their other functions, such as cytokine secretion (Figure 6E). Overall, these results showed that PRAK was essential for neutrophil NET formation in bacterial killing following CLP-induced sepsis.

## Disscussion

In this study, we explored whether PRAK played a role in regulating both human and murine NET formation. Our data showed that PRAK dysfunctional human neutrophils and PRAK-deficient murine neutrophils displayed defective NET formation. PRAK deficiency could bring about impaired NET-mediated antibacterial activity as well as shortened the survival in mice with CLP-induced sepsis. Furthermore, our results revealed a novel mechanism that intracellular ROS regulated by PRAK could maintain NETosis and apoptosis balance in neutrophils. Rational PRAK-ROS can suppress apoptosis through mTOR-autophagy activation and then left a chance to initiate NET formation.

NETosis and apoptosis are two distinct ways of cell death with different morphological features and signaling pathways (42, 43). NETosis is characterized by decondensed chromatin and membrane split, and this process does not depend on caspase enzymes (18). While the features of apoptosis are condensation of chromatin and caspase activity (44). Usually, when encountering infection, neutrophils will use NETosis which is the preferred method to initiate to clean up bacteria (6). However, this choice could be interrupted. Remijsen et al. observed that when autophagy or NADPH oxidase activity was blocked by wortmannin or DPI in PMA-stimulated neutrophils, apoptotic features would become predominant (15). Mechanistically, NET formation depended on NADPH oxidase activation mediated by Raf-MEK-ERK pathway and several anti-apoptotic proteins such as Mcl-1 would be upregulated to inhibit apoptosis during NETosis (39). However, high ROS which is produced by NADPH also can damage NET formation and lead neutrophils to apoptosis, although the specific mechanism is unknown (15, 40). In our data, PRAK played an important role in NET formation by influencing ROS release. Disabled PRAK could not regulate ROS correctly and thereafter give rise to higher caspase 3 expression and accumulation of autophagy. Finally, PRAK deficiency resulted in much elevated apoptotic cells and limited NETotic neutrophils. Therefore, it can be assumed that the balance between NETosis and apoptosis is controlled by the ROS production, since it could be rescued by lower ROS production caused by hypoxia. When facing bacterial infection, neutrophils which have proper ROS content will keep the caspase enzymes inactive and then go to NETosis to clean up the bacteria. However, neutrophils which lose the reasonable ROS releasing by any factor will face active autophagy formation and switch the death way from NETs to apoptosis. Here, we proposed a novel candidate, PRAK, which can feel the oxidative stress and regulate the ROS releasing, may be the master molecular switch to regulate the NET-apoptosis axis of neutrophils.

mTOR has been reported to be an inhibitor of apoptosis (45). Interestingly, Mclnturff et al. reported that blockade of mTOR inhibited LPS-induced NET formation through translational control of HIF-1α (24). How mTOR and autophagy could interfere with NETosis is still unknown. In the present study, we demonstrated that PRAK dysfunctional neutrophils showed attenuation of the mTOR activity and enhanced autophagy formation. Alexander et al. reported that ROS was suggested to repress mTORC1 in a dose- and time-dependent manner when cells were stimulated with H_2_O_2_ (46). And we propose that upon oxidative stress, PRAK could boost mTOR pathway signal by inhibiting ROS production. This hypothesis somehow may contradict with certain previous studies which showed that PRAK directly promoted the phosphorylation of Ras homolog enriched in brain (Rheb) in MEF cells, thereby inhibiting Rheb-mediated mTORC1 activation during energy starvation (47). For this, we suspected that the relationship between PRAK and mTOR might be distinct in various cell types. Or PRAK, as a regulator of ROS under oxidative stress, may regulate mTOR in NET formation through other pathways.

Autophagy is necessary for NET formation. As we mentioned above, defects in autophagy by autophagy inhibitors can significantly inhibit the formation of autophagic vacuolization, thereby suppressing NET formation (15, 48). Park et al. reported that neutrophils isolated from surviving sepsis patients showed increased autophagic vacuoles and NET formation than healthy neutrophils (21). Conversely, rapamycin used as autophagy agonist can reduce LPS-induced NET formation (24). In our opinion, autophagy may play a complicated role in the NET process. Up till now, almost all the studies on NET formation and autophagy were based on stimulation *in vitro* and when there was a lack of autophagy, NET formation was blocked. No data shows what will happen in neutrophils if ROS causes autophagy to be overwhelming. Here we found that the existence of PRAK inhibitor would straighten autophagy influx and excessive autophagy can ultimately terminate the NETosis, sharply hurting the bacterial cleaning of neutrophils. Neutrophils treated with PRAK inhibitor induced the conversion of LC3-I to LC3-II, and enhanced the degradation of p62 in response to PMA stimulation. Our data also showed that hypoxia inhibited the release of ROS and thereby limited over-activity of autophagy. PRAK dysfunction may be associated with elevated caspase-dependent apoptosis, owing to over-activity of autophagy formation caused by the enhanced mTOR repression. Due to the firm relationship between PRAK and autophagy inducer ROS, we validated that PRAK dysfunction could lead to excessive ROS, and ROS promote autophagy formation and finally turn to apoptosis but not NETosis. Therefore, our current study complements recent reports on the mechanism how autophagy can control the apoptosis and NETosis in human and mouse.

In summary, PRAK could control the ROS releasing, maintain the mTOR signal, limit the over-activation of autophagy and prevent apoptosis during NET formation. Accordingly, PRAK may be the key controller to precisely adjust the balance between apoptosis and NET formation under pathogen stress. Therefore, by redirecting NETosis, PRAK inhibitor may provide a potential therapeutic strategy, such as a combination with DNase reagent to treat inflammatory and autoimmune diseases (49, 50).

## Ethics Statement

All the animal procedures were conformed to the Chinese Council on Animal Care Guidelines and the study was approved by the Ethics Committee of Peking University Health Science Center. Peripheral blood used in the research was from healthy volunteers. The research was approved by the Ethics Committee of Peking University People's Hospital, and performed in accordance with the ethical standards of Declaration of Helsinki.

## Author Contributions

YaW, YZha, and WW designed the project. YaW and WW did the experiment and wrote the manuscript. YuW, YZho, and JW contributed to establish the CLP model and analyze the data. CL collected data. LM raised the *Prak*^flox/flox^ and *Prak*^flox/flox^ *Lyz2*-Cre mice.

### Conflict of Interest Statement

The authors declare that the research was conducted in the absence of any commercial or financial relationships that could be construed as a potential conflict of interest.

## Supplementary Material

Figure S1 PRAK inhibitor affects NET formation with a dose-dependent manner. **(A)** NET formation was measured by cell-free DNA concentration according to the manufacturer's instructions (*n* = 5, ^**^*P* < 0.001, paired *t*-test). **(B)** Neutrophils were pretreated with 0–10 μM PRAK inhibitor and further stimulated with PMA for 4 h. NETs were stained with anti-MPO (MPO; green) and DNA (Hoechst 33342; blue). **(C)** NET formation was quantified with fluorescence microscopic analysis. Results are presented as the percentage of control (*n* = 5, ^*^*P* < 0.05, ^***^*P* < 0.001, one-way ANOVA). **(D)** The apoptotic neutrophils incubated with 0–10 μM PRAK inhibitor for 12 h were detected via flow cytometry using Annexin V/7AAD. **(E)** Representative images and of NE translocation are shown (NE, green; DNA, blue). Scale bars = 5 μm.

Figure S2 PRAK inhibitor suppresses NET-mediated extracellular bacterial killing. **(A,B)** NET-mediated bacterial killing was determined as described in Methods. Representative images of extracellular residual bacteria are shown.

Figure S3 Effect of PRAK inhibitor on ERK pathway. **(A)** Western blot analysis of phospho-ERK in neutrophils stimulated with PMA for 30 min with or without PRAK inhibitor pretreatment.

Figure S4 Hypoxic environment (5% O2) rescues NET-mediated bacterial killing and NE translocation in PRAK dysfunctional neutrophils. **(A,B)** Representative images and quantification of NE translocation are shown. Scale bar = 7.5 μm. ^*^*P* < 0.05, ^**^*P* < 0.01. **(C)** Representative images of extracellular residual bacteria are shown. **(D)** Results are shown as the mean percentage of bacterial killing ± SD. ^*^*P* < 0.05, ^**^*P* < 0.01. The experiments were repeated 5 times. *P-*values were obtained by two-way ANOVA.

Figure S5 PRAK has on effect on NOX-independent NETs. Neutrophils were pretreated with or without PRAK inhibitor (5 μM) for 1 h, and then stimulated with LPS (25 μg/ml) or ionomycin (5 μM) for 4 h at 37°C and 5% CO_2_. NETs were stained with anti-MPO (MPO; green) and DNA (Hoechst 33342; blue). **(A,B)** Representative images and quantification of LPS-induced NETs are shown. Scale bar = 10 μm. *n* = 5, ^**^*P* < 0.01, paired *t*-test. **(C,D)** Representative images and quantification of ionomycin-induced NETs are shown. Scale bar = 10 μm. *n* = 5, no significant, paired *t*-test.

Figure S6 PRAK-deficient neutrophils show reduced NET formation in mice with CLP-induced sepsis. **(A)** Amount of cf-DNA in the plasma at 6 h after CLP (*n* = 5, ^*^*P* < 0.05, *t*-test).

## Abbreviations

NETs: neutrophil extracellular traps
ROS: reactive oxygen species
PMA: Phorbol 12-myristate 13-acetate
NE: neutrophil elastase
MPO: myeloperoxidase
mTOR: mammalian target of rapamycin
CLP: cecal ligation and puncture
WT: wild-type
KO: knockout
cf-DNA: cell-free DNA.

## References

[1] Nathan C. Neutrophils and immunity: challenges and opportunities. Nat Rev Immunol. (2006) 6:173–82. 10.1038/nri1785 16498448

[2] Amulic B Cazalet C Hayes GL Metzler KD Zychlinsky A. Neutrophil function: from mechanisms to disease. Annu Rev Immunol. (2012) 30:459–89. 10.1146/annurev-immunol-020711-074942 22224774

[3] Eyles JL Roberts AW Metcalf D Wicks IP. Granulocyte colony-stimulating factor and neutrophils–forgotten mediators of inflammatory disease. Nat Clin Pract Rheumatol. (2006) 2:500–10. 10.1038/ncprheum0291 16951705

[4] Brinkmann V Zychlinsky A. Neutrophil extracellular traps: is immunity the second function of chromatin? J Cell Biol. (2012) 198:773–83. 10.1083/jcb.201203170 22945932 PMC3432757

[5] Brinkmann V Reichard U Goosmann C Fauler B Uhlemann Y Weiss DS. Neutrophil extracellular traps kill bacteria. Science. (2004) 303:1532–5. 10.1126/science.1092385 15001782

[6] Clark SR Ma AC Tavener SA McDonald B Goodarzi Z Kelly MM. Platelet TLR4 activates neutrophil extracellular traps to ensnare bacteria in septic blood. Nat Med. (2007) 13:463–9. 10.1038/nm1565 17384648

[7] Urban CF Reichard U Brinkmann V Zychlinsky A. Neutrophil extracellular traps capture and kill Candida albicans yeast and hyphal forms. Cell Microbiol. (2006) 8:668–76. 10.1111/j.1462-5822.2005.00659.x 16548892

[8] Abi Abdallah DS Lin C Ball CJ King MR Duhamel GE Denkers EY. Toxoplasma gondii triggers release of human and mouse neutrophil extracellular traps. Infect Immun. (2012) 80:768–77. 10.1128/IAI.05730-11 22104111 PMC3264325

[9] Kaplan MJ Radic M. Neutrophil extracellular traps: double-edged swords of innate immunity. J Immunol. (2012) 189:2689–95. 10.4049/jimmunol.1201719 22956760 PMC3439169

[10] Knight JS Carmona-Rivera C Kaplan MJ. Proteins derived from neutrophil extracellular traps may serve as self-antigens and mediate organ damage in autoimmune diseases. Front Immunol. (2012) 3:380. 10.3389/fimmu.2012.00380 23248629 PMC3521997

[11] Sur Chowdhury C Giaglis S Walker UA Buser A Hahn S Hasler P. Enhanced neutrophil extracellular trap generation in rheumatoid arthritis: analysis of underlying signal transduction pathways and potential diagnostic utility. Arthritis Res Ther. (2014) 16:R122. 10.1186/ar4579 24928093 PMC4229860

[12] Leffler J Martin M Gullstrand B Tyden H Lood C Truedsson L. Neutrophil extracellular traps that are not degraded in systemic lupus erythematosus activate complement exacerbating the disease. J Immunol. (2012) 188:3522–31. 10.4049/jimmunol.1102404 22345666

[13] Sangaletti S Tripodo C Chiodoni C Guarnotta C Cappetti B Casalini P. Neutrophil extracellular traps mediate transfer of cytoplasmic neutrophil antigens to myeloid dendritic cells toward ANCA induction and associated autoimmunity. Blood. (2012) 120:3007–18. 10.1182/blood-2012-03-416156 22932797

[14] Mitroulis I Kambas K Chrysanthopoulou A Skendros P Apostolidou E Kourtzelis I. Neutrophil extracellular trap formation is associated with IL-1beta and autophagy-related signaling in gout. PLoS One. (2011) 6:e29318. 10.1371/journal.pone.0029318 22195044 PMC3241704

[15] Remijsen Q Vanden Berghe T Wirawan E Asselbergh B Parthoens E De Rycke R. Neutrophil extracellular trap cell death requires both autophagy and superoxide generation. Cell Res. (2011) 21:290–304. 10.1038/cr.2010.150 21060338 PMC3193439

[16] Stoiber W Obermayer A Steinbacher P Krautgartner WD. The Role of Reactive Oxygen Species (ROS) in the Formation of Extracellular Traps (ETs) in humans. Biomolecules. (2015) 5:702–23. 10.3390/biom5020702 25946076 PMC4496692

[17] Parker H Dragunow M Hampton MB Kettle AJ Winterbourn CC. Requirements for NADPH oxidase and myeloperoxidase in neutrophil extracellular trap formation differ depending on the stimulus. J Leukoc Biol. (2012) 92:841–9. 10.1189/jlb.1211601 22802447

[18] Fuchs TA Abed U Goosmann C Hurwitz R Schulze I Wahn V. Novel cell death program leads to neutrophil extracellular traps. J Cell Biol. (2007) 176:231–41. 10.1083/jcb.200606027 17210947 PMC2063942

[19] Keshari RS Verma A Barthwal MK Dikshit M. Reactive oxygen species-induced activation of ERK and p38 MAPK mediates PMA-induced NETs release from human neutrophils. J Cell Biochem. (2013) 114:532–40. 10.1002/jcb.24391 22961925

[20] Leshner M Wang S Lewis C Zheng H Chen XA Santy L. PAD4 mediated histone hypercitrullination induces heterochromatin decondensation and chromatin unfolding to form neutrophil extracellular trap-like structures. Front Immunol. (2012) 3:307. 10.3389/fimmu.2012.00307 23060885 PMC3463874

[21] Park SY Shrestha S Youn YJ Kim JK Kim SY Kim HJ. Autophagy primes neutrophils for neutrophil extracellular trap formation during sepsis. Am J Respir Crit Care Med. (2017) 196:577–89. 10.1164/rccm.201603-0596OC 28358992

[22] Pieterse E Rother N Yanginlar C Hilbrands LB van der Vlag J. Neutrophils discriminate between lipopolysaccharides of different bacterial sources and selectively release neutrophil extracellular traps. Front Immunol. (2016) 7:484. 10.3389/fimmu.2016.00484 27867387 PMC5095130

[23] Jung CH Jun CB Ro SH Kim YM Otto NM Cao J. ULK-Atg13-FIP200 complexes mediate mTOR signaling to the autophagy machinery. Mol Biol Cell. (2009) 20:1992–2003. 10.1091/mbc.e08-12-1249 19225151 PMC2663920

[24] McInturff AM Cody MJ Elliott EA Glenn JW Rowley JW Rondina MT. Mammalian target of rapamycin regulates neutrophil extracellular trap formation via induction of hypoxia-inducible factor 1 alpha. Blood. (2012) 120:3118–25. 10.1182/blood-2012-01-405993 22919032 PMC3471519

[25] Douda DN Khan MA Grasemann H Palaniyar N. SK3 channel and mitochondrial ROS mediate NADPH oxidase-independent NETosis induced by calcium influx. Proc Natl Acad Sci USA. (2015) 112:2817–22. 10.1073/pnas.1414055112 25730848 PMC4352781

[26] Arai Y Nishinaka Y Arai T Morita M Mizugishi K Adachi S. Uric acid induces NADPH oxidase-independent neutrophil extracellular trap formation. Biochem Biophys Res Commun. (2014) 443:556–61. 10.1016/j.bbrc.2013.12.007 24326071

[27] New L Jiang Y Zhao M Liu K Zhu W Flood LJ. PRAK, a novel protein kinase regulated by the p38 MAP kinase. EMBO J. (1998) 17:3372–84. 10.1093/emboj/17.12.3372 9628874 PMC1170675

[28] Wang C Wang H Chang DY Hao J Zhao MH Chen M. High mobility group box 1 contributes to anti-neutrophil cytoplasmic antibody-induced neutrophils activation through receptor for advanced glycation end products (RAGE) and Toll-like receptor 4. Arthritis Res Ther. (2015) 17:64. 10.1186/s13075-015-0587-4 25889374 PMC4382936

[29] Mohammed BM Fisher BJ Kraskauskas D Farkas D Brophy DF Fowler AA III. Vitamin C: a novel regulator of neutrophil extracellular trap formation. Nutrients. (2013) 5:3131–51. 10.3390/nu5083131 23939536 PMC3775246

[30] Brinkmann V Goosmann C Kuhn LI Zychlinsky A. Automatic quantification of *in vitro* NET formation. Front Immunol. (2012) 3:413. 10.3389/fimmu.2012.00413 23316198 PMC3540390

[31] Brinkmann V Laube B Abu Abed U Goosmann C Zychlinsky A. Neutrophil extracellular traps: how to generate and visualize them. J Vis Exp. (2010) e1724. 10.3791/1724 20182410 PMC3125121

[32] Rittirsch D Huber-Lang MS Flierl MA Ward PA. Immunodesign of experimental sepsis by cecal ligation and puncture. Nat Protoc. (2009) 4:31–6. 10.1038/nprot.2008.214 19131954 PMC2754226

[33] Papayannopoulos V Metzler KD Hakkim A Zychlinsky A. Neutrophil elastase and myeloperoxidase regulate the formation of neutrophil extracellular traps. J Cell Biol. (2010) 191:677–91. 10.1083/jcb.201006052 20974816 PMC3003309

[34] Metzler KD Goosmann C Lubojemska A Zychlinsky A Papayannopoulos VA. myeloperoxidase-containing complex regulates neutrophil elastase release and actin dynamics during NETosis. Cell Rep. (2014) 8:883–96. 10.1016/j.celrep.2014.06.044 25066128 PMC4471680

[35] Branzk N Papayannopoulos V. Molecular mechanisms regulating NETosis in infection and disease. Semin Immunopathol. (2013) 35:513–30. 10.1007/s00281-013-0384-6 23732507 PMC3685711

[36] Branzk N Lubojemska A Hardison SE Wang Q Gutierrez MG Brown GD. Neutrophils sense microbe size and selectively release neutrophil extracellular traps in response to large pathogens. Nat Immunol. (2014) 15:1017–25. 10.1038/ni.2987 25217981 PMC4236687

[37] Douda DN Yip L Khan MA Grasemann H Palaniyar N. Akt is essential to induce NADPH-dependent NETosis and to switch the neutrophil death to apoptosis. Blood. (2014) 123:597–600. 10.1182/blood-2013-09-526707 24458280

[38] Jung CH Ro SH Cao J Otto NM Kim DH. mTOR regulation of autophagy. FEBS Lett. (2010) 584:1287–95. 10.1016/j.febslet.2010.01.017 20083114 PMC2846630

[39] Hakkim A Fuchs TA Martinez NE Hess S Prinz H Zychlinsky A. Activation of the Raf-MEK-ERK pathway is required for neutrophil extracellular trap formation. Nat Chem Biol. (2011) 7:75–7. 10.1038/nchembio.496 21170021

[40] Tsurubuchi T Aratani Y Maeda N Koyama H. Retardation of early-onset PMA-induced apoptosis in mouse neutrophils deficient in myeloperoxidase. J Leukoc Biol. (2001) 70:52–8. 10.1189/jlb.70.1.52 11435485

[41] McDonald B Urrutia R Yipp BG Jenne CN Kubes P. Intravascular neutrophil extracellular traps capture bacteria from the bloodstream during sepsis. Cell Host Microbe. (2012) 12:324–33. 10.1016/j.chom.2012.06.011 22980329

[42] Radic M. Clearance of apoptotic bodies, NETs, and biofilm DNA: implications for autoimmunity. Front Immunol. (2014) 5:365. 10.3389/fimmu.2014.00365 25126089 PMC4115591

[43] Remijsen Q Kuijpers TW Wirawan E Lippens S Vandenabeele P Vanden Berghe T. Dying for a cause: NETosis, mechanisms behind an antimicrobial cell death modality. Cell Death Differ. (2011) 18:581–8. 10.1038/cdd.2011.1 21293492 PMC3131909

[44] Iglesias-Guimarais V Gil-Guinon E Sanchez-Osuna M Casanelles E Garcia-Belinchon M Comella JX. Chromatin collapse during caspase-dependent apoptotic cell death requires DNA fragmentation factor, 40-kDa subunit-/caspase-activated deoxyribonuclease-mediated 3'-OH single-strand DNA breaks. J Biol Chem. (2013) 288:9200–15. 10.1074/jbc.M112.411371 23430749 PMC3610992

[45] Castedo M Ferri KF Kroemer G. Mammalian target of rapamycin (mTOR): pro- and anti-apoptotic. Cell Death Differ. (2002) 9:99–100. 10.1038/sj.cdd.4400978 11840159

[46] Alexander A Kim J Walker CL. ATM engages the TSC2/mTORC1 signaling node to regulate autophagy. Autophagy. (2010) 6:672–3. 10.4161/auto.6.5.12509 20581436 PMC3259740

[47] Zheng M Wang YH Wu XN Wu SQ Lu BJ Dong MQ. Inactivation of Rheb by PRAK-mediated phosphorylation is essential for energy-depletion-induced suppression of mTORC1. Nat Cell Biol. (2011) 13:263–72. 10.1038/ncb2168 21336308 PMC3070924

[48] Tang S Zhang Y Yin SW Gao XJ Shi WW Wang Y. Neutrophil extracellular trap formation is associated with autophagy-related signalling in ANCA-associated vasculitis. Clin Exp Immunol. (2015) 180:408–18. 10.1111/cei.12589 25644394 PMC4449769

[49] Hakkim A Furnrohr BG Amann K Laube B Abed UA Brinkmann V. Impairment of neutrophil extracellular trap degradation is associated with lupus nephritis. Proc Natl Acad Sci USA. (2010) 107:9813–8. 10.1073/pnas.0909927107 20439745 PMC2906830

[50] Barnado A Crofford LJ Oates JC. At the bedside: neutrophil extracellular traps (NETs) as targets for biomarkers and therapies in autoimmune diseases. J Leukoc Biol. (2016) 99:265–78. 10.1189/jlb.5BT0615-234R 26658004 PMC6608010

