# Supplementary figures and images for: PRAK Is Required for the Formation of Neutrophil Extracellular Traps
Source: Front Immunol. 2019 Jun 4;10:1252. doi: 10.3389/fimmu.2019.01252 (PMC6559312; doi:10.3389/fimmu.2019.01252)

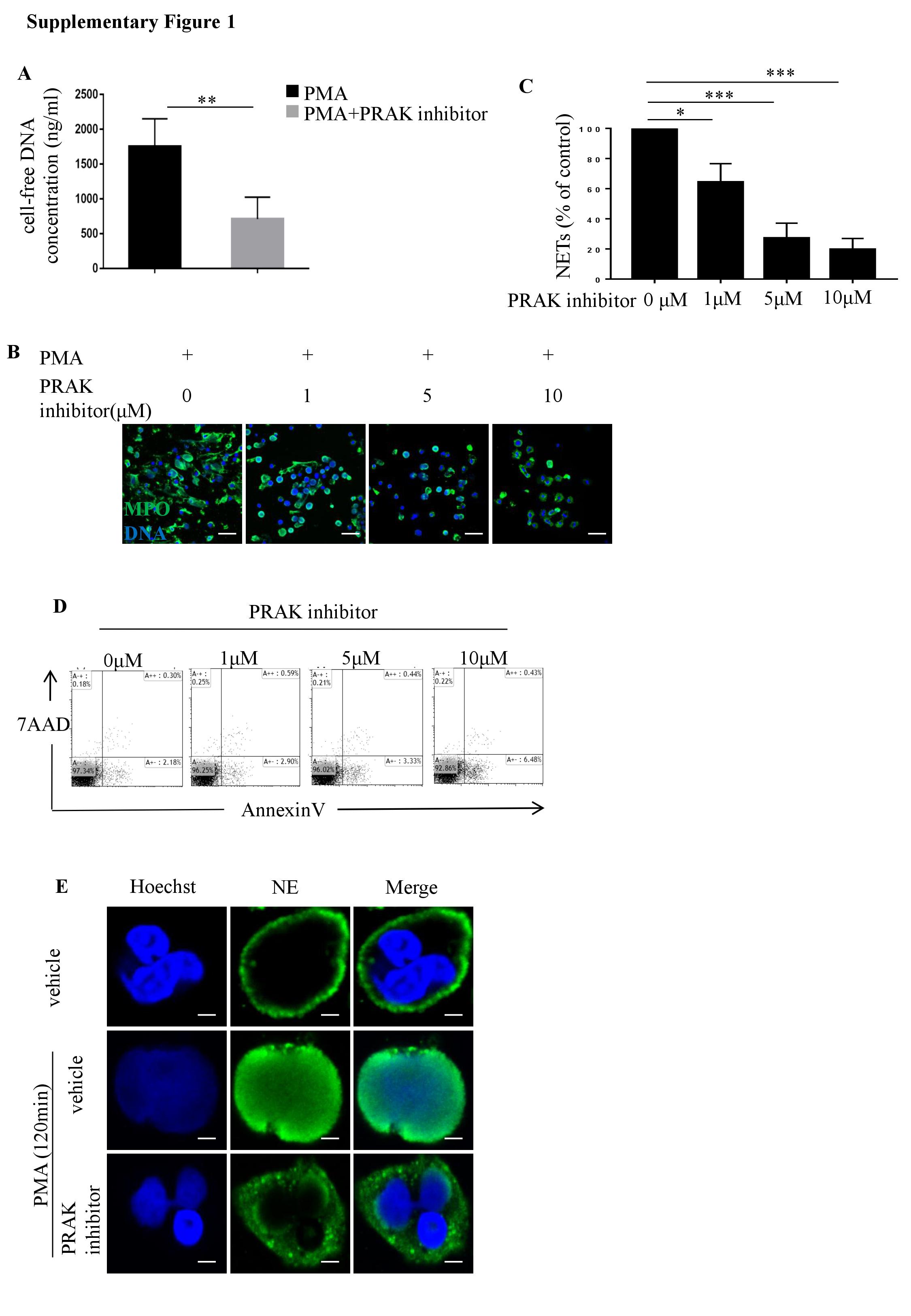

Supplement: Figure S1 — PRAK inhibitor affects NET formation with a dose-dependent manner. (A) NET formation was measured by cell-free DNA concentration according to the manufacturer's instructions (n = 5, **P < 0.001, paired t-test). (B) Neutrophils were pretreated with 0–10 μM PRAK inhibitor and further stimulated with PMA for 4 h. NETs were stained with anti-MPO (MPO; green) and DNA (Hoechst 33342; blue). (C) NET formation was quantified with fluorescence microscopic analysis. Results are presented as the percentage of control (n = 5, *P < 0.05, ***P < 0.001, one-way ANOVA). (D) The apoptotic neutrophils incubated with 0–10 μM PRAK inhibitor for 12 h were detected via flow cytometry using Annexin V/7AAD. (E) Representative images and of NE translocation are shown (NE, green; DNA, blue). Scale bars = 5 μm. [file Image_1.JPEG]

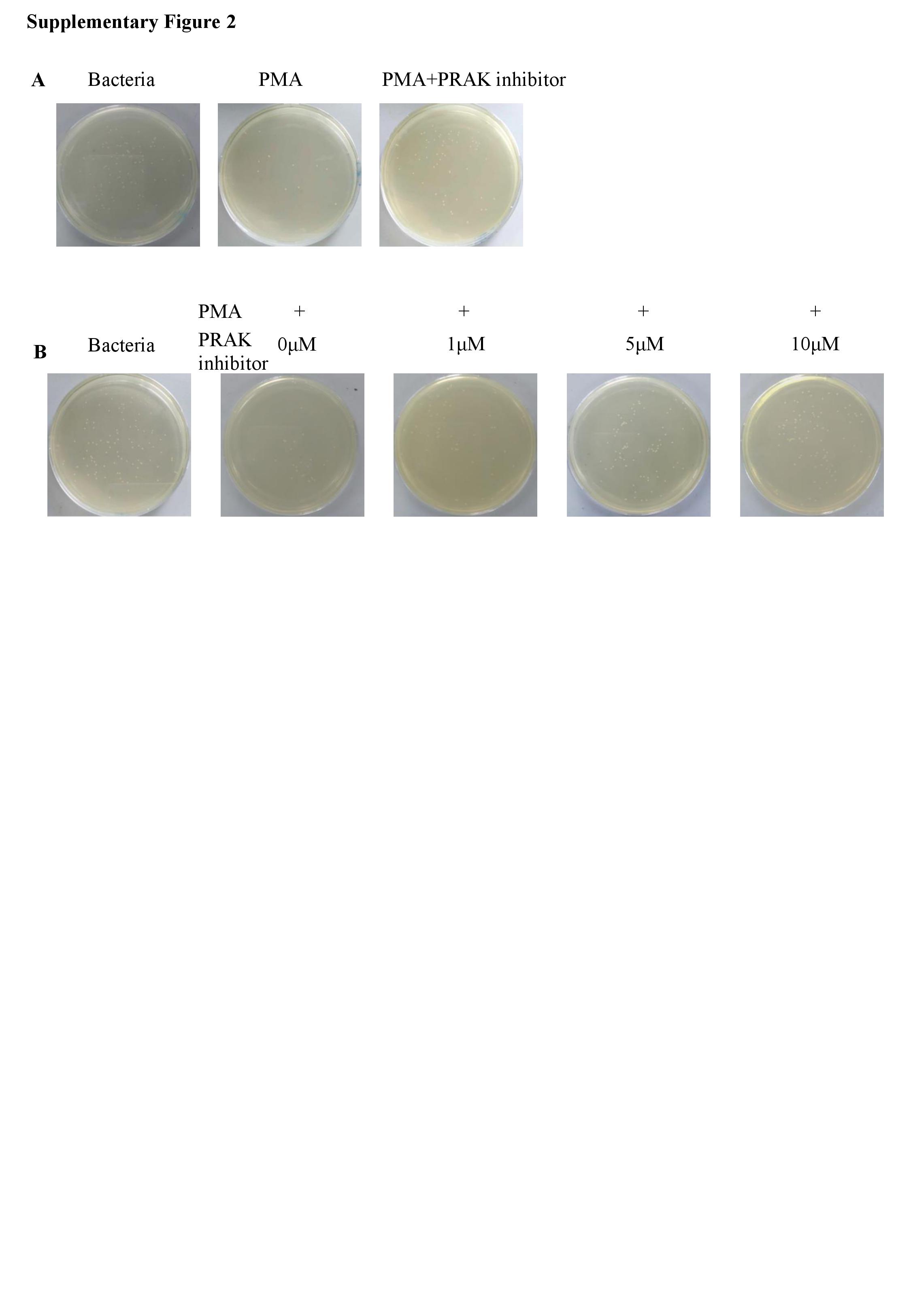

Supplement: Figure S2 — PRAK inhibitor suppresses NET-mediated extracellular bacterial killing. (A,B) NET-mediated bacterial killing was determined as described in Methods. Representative images of extracellular residual bacteria are shown. [file Image_2.JPEG]

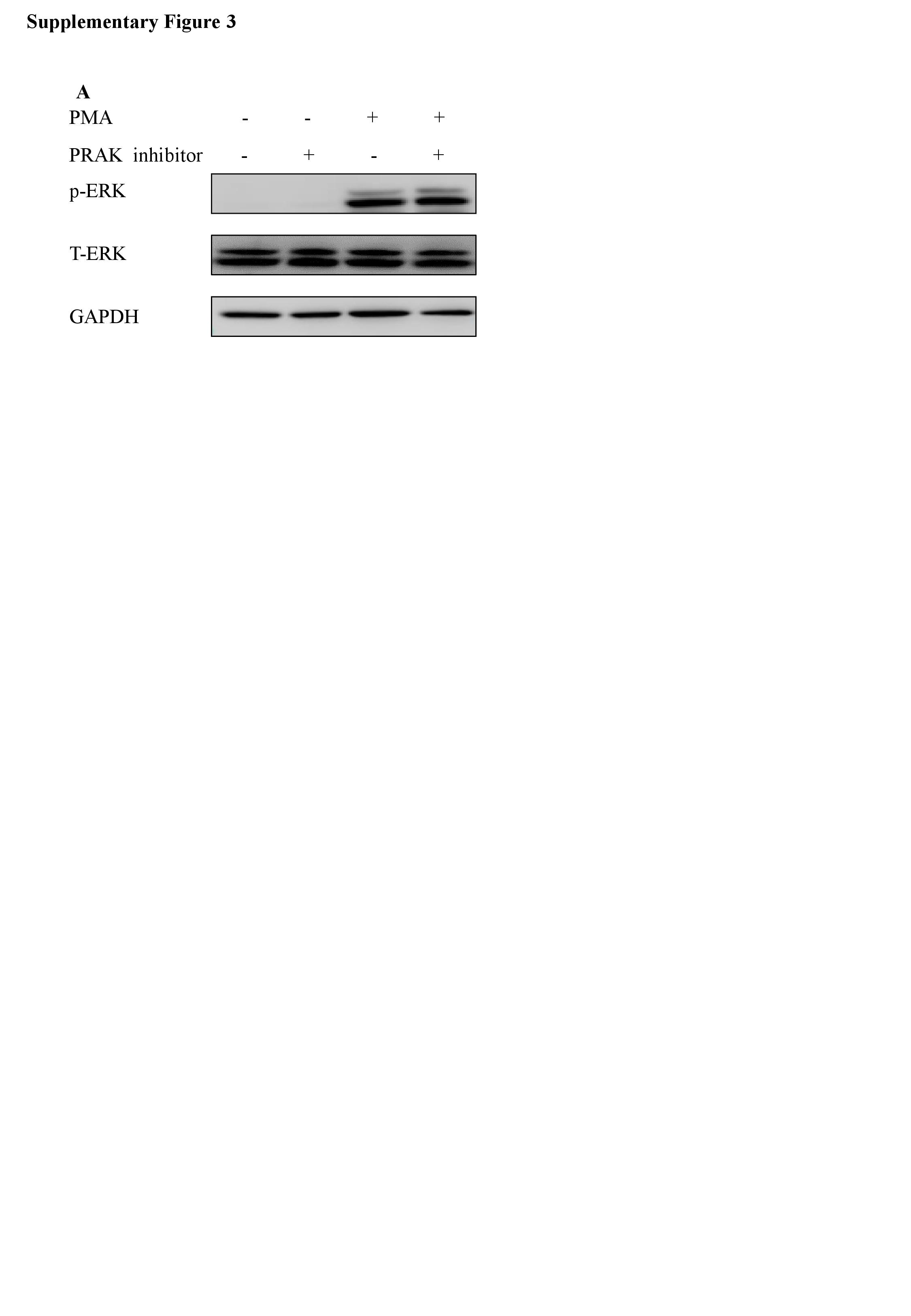

Supplement: Figure S3 — Effect of PRAK inhibitor on ERK pathway. (A) Western blot analysis of phospho-ERK in neutrophils stimulated with PMA for 30 min with or without PRAK inhibitor pretreatment. [file Image_3.JPEG]

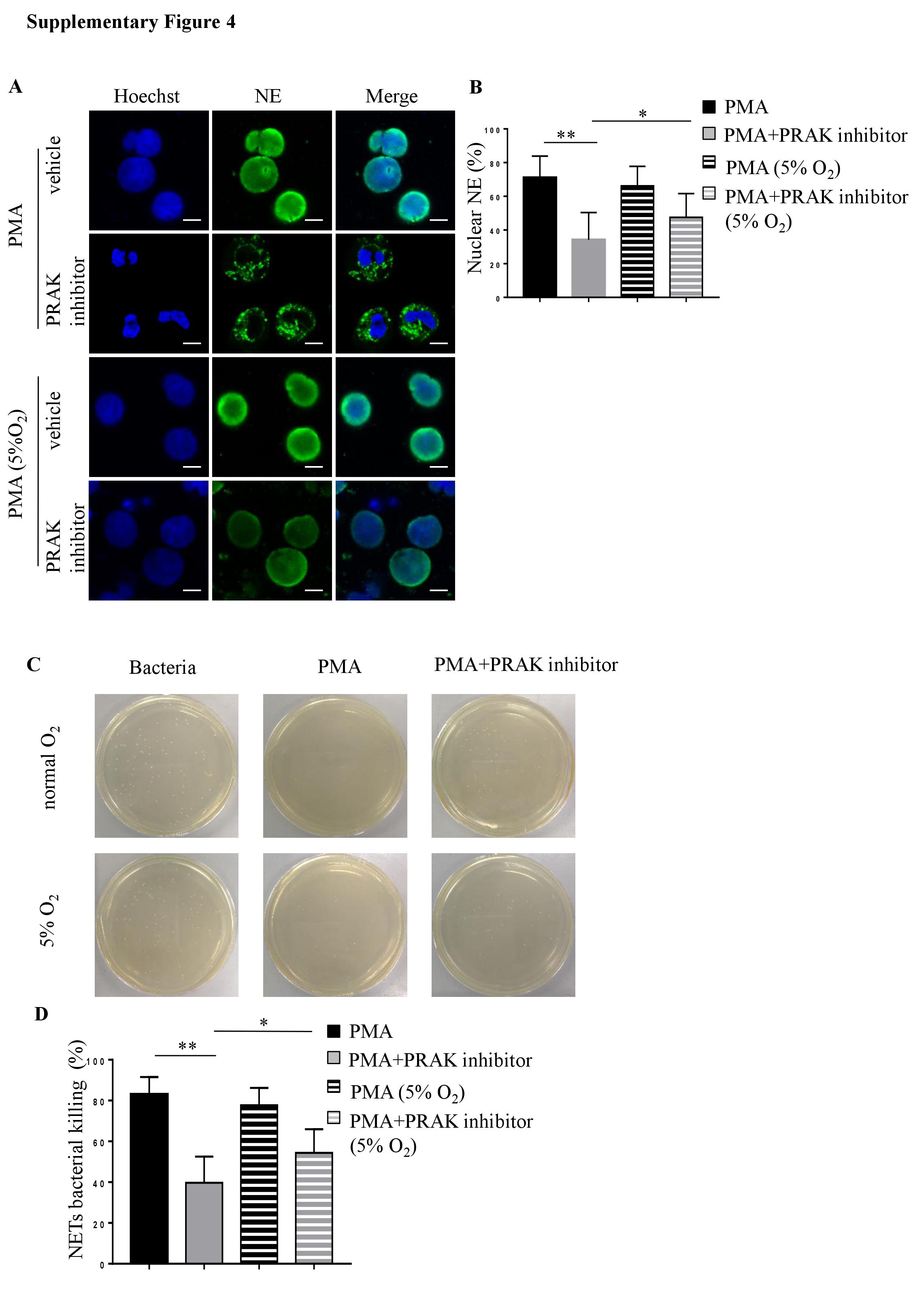

Supplement: Figure S4 — Hypoxic environment (5% O2) rescues NET-mediated bacterial killing and NE translocation in PRAK dysfunctional neutrophils. (A,B) Representative images and quantification of NE translocation are shown. Scale bar = 7.5 μm. *P < 0.05, **P < 0.01. (C) Representative images of extracellular residual bacteria are shown. (D) Results are shown as the mean percentage of bacterial killing ± SD. *P < 0.05, **P < 0.01. The experiments were repeated 5 times. P-values were obtained by two-way ANOVA. [file Image_4.JPEG]

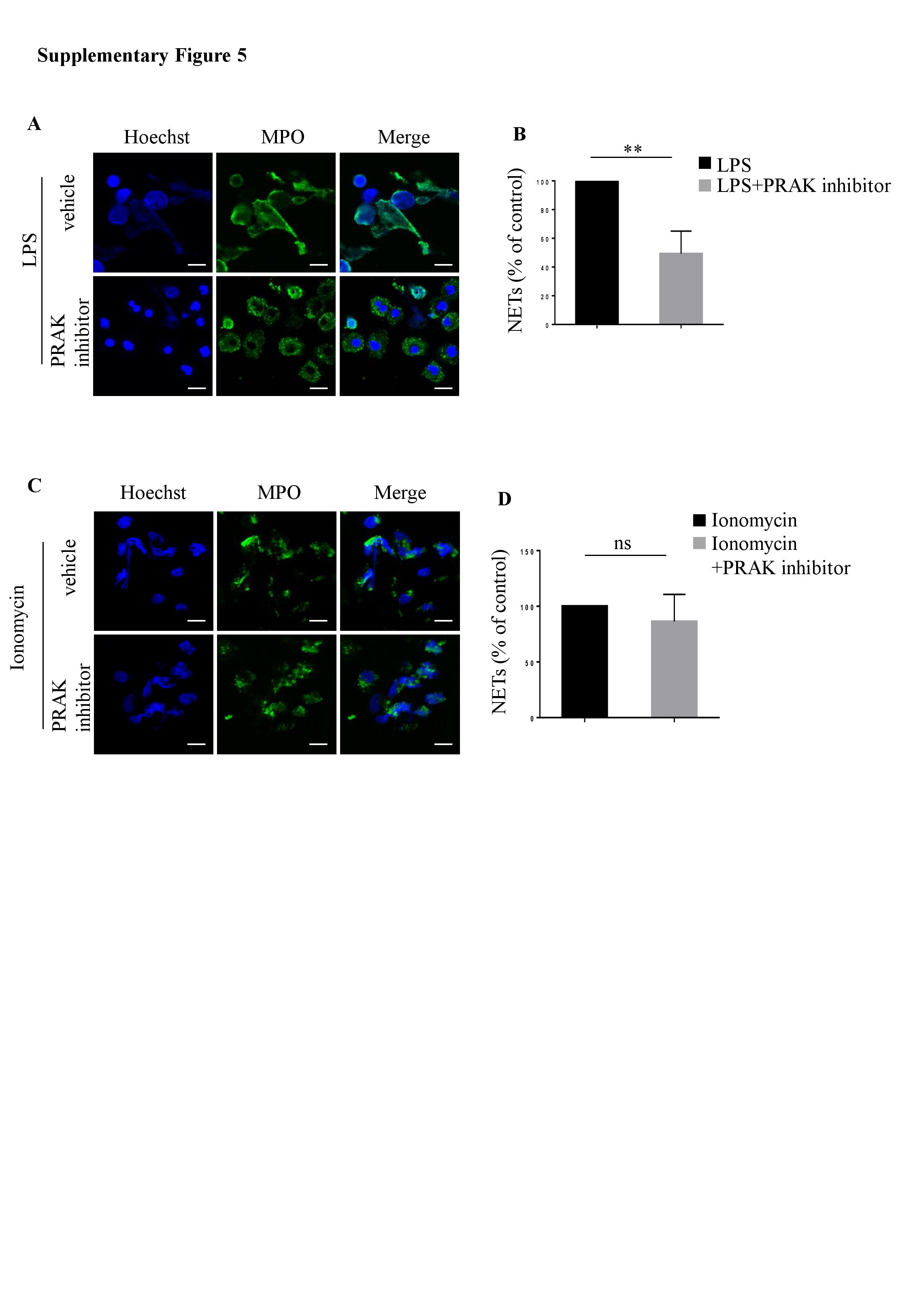

Supplement: Figure S5 — PRAK has on effect on NOX-independent NETs. Neutrophils were pretreated with or without PRAK inhibitor (5 μM) for 1 h, and then stimulated with LPS (25 μg/ml) or ionomycin (5 μM) for 4 h at 37°C and 5% CO2. NETs were stained with anti-MPO (MPO; green) and DNA (Hoechst 33342; blue). (A,B) Representative images and quantification of LPS-induced NETs are shown. Scale bar = 10 μm. n = 5, **P < 0.01, paired t-test. (C,D) Representative images and quantification of ionomycin-induced NETs are shown. Scale bar = 10 μm. n = 5, no significant, paired t-test. [file Image_5.jpg]

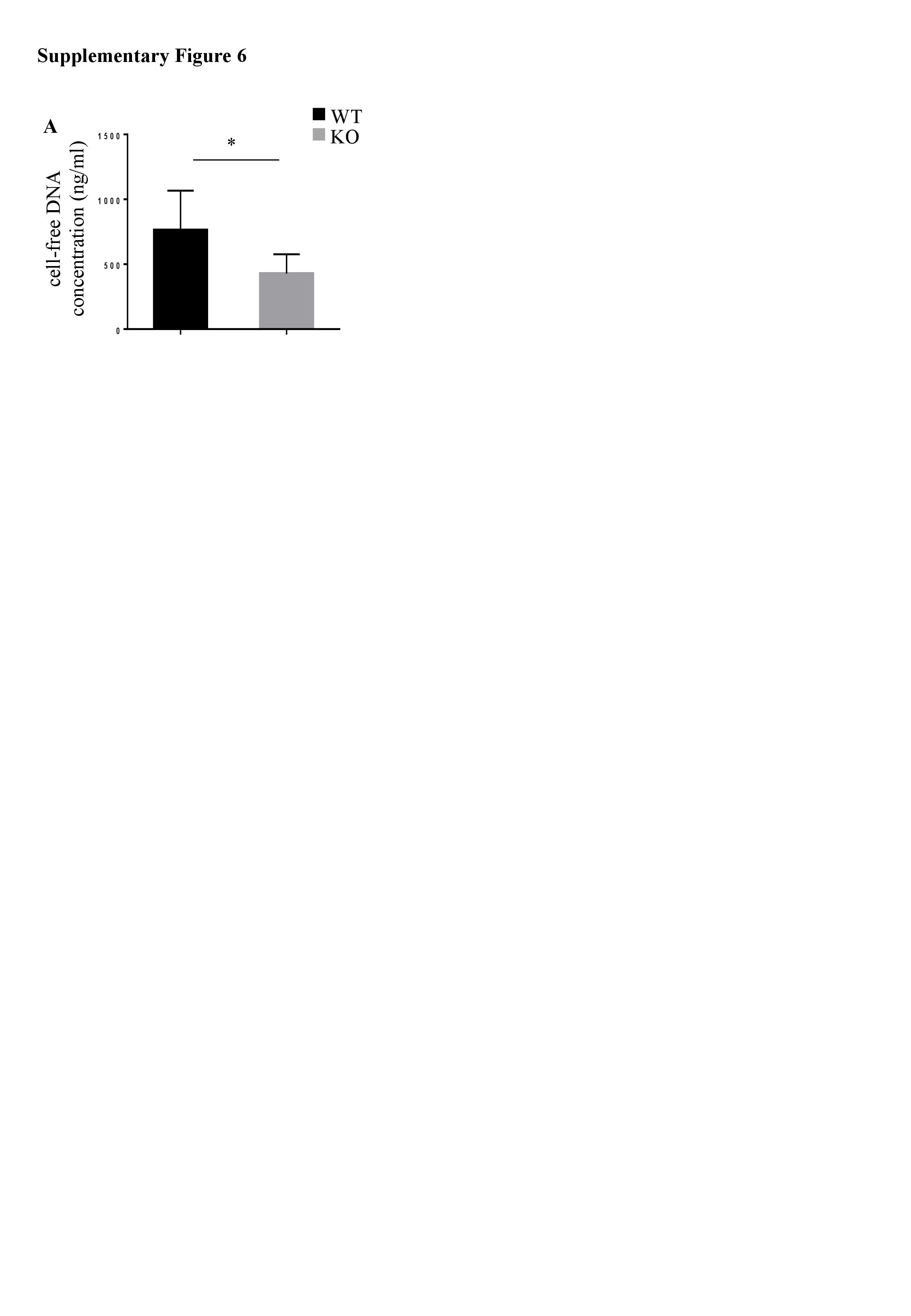

Supplement: Figure S6 — PRAK-deficient neutrophils show reduced NET formation in mice with CLP-induced sepsis. (A) Amount of cf-DNA in the plasma at 6 h after CLP (n = 5, *P < 0.05, t-test). [file Image_6.JPEG]
